# Validation of a Stability-Indicating RP-HPLC Method for Determination of l-Carnitine in Tablets

**DOI:** 10.1155/2014/481059

**Published:** 2014-10-22

**Authors:** Roghaieh Khoshkam, Minoo Afshar

**Affiliations:** Department of Pharmaceutics, Faculty of Pharmacy, Islamic Azad University (IAUPS), Pharmaceutical Sciences Branch, Tehran 193956466, Iran

## Abstract

A rapid and stability-indicating RP-HPLC method was developed for determination of l-carnitine in tablets. The separation was based on a C18 analytical column using a mobile phase which consisted of 0.05 M phosphate buffer (pH = 3): ethanol (99 : 1), including 0.56 mg/mL of sodium 1-heptanesulfonate. Column temperature was set at 50°C and quantitation was achieved by UV detection at 225 nm. In forced degradation studies, the drug was subjected to oxidation, hydrolysis, photolysis, and heat. Among the different stress conditions, the exposure to acidic and basic conditions was found to be an important adverse stability factor. The method was validated for specificity, selectivity, linearity, precision, accuracy, and robustness. The applied procedure was found to be linear in l-carnitine concentration range of 84.74–3389.50 *µ*g/mL (*r* ^2^ = 0.9997). Precision was evaluated by replicate analysis in which relative standard deviation (RSD) values for areas were found below 2.0%. The recoveries obtained (100.83%–101.54%) ensured the accuracy of the developed method. The expanded uncertainty (3.14%) of the method was also estimated from method validation data. Accordingly, the proposed validated and rapid procedure was proved to be suitable for routine analyzing and stability studies of l-carnitine in tablets.

## 1. Introduction

**Figure 1:**
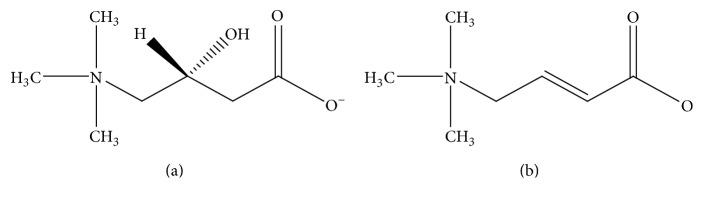
Molecular structures of l-carnitine (a) and crotonoylbetaine (b).

l-Carnitine ((R)-3-carboxy-2-hydroxy-N,N,N-trimethyl-1-propaminium hydroxide inner salt, Figure 1(a)) is a vitamin-like amino acid derivative, which is an essential factor in fatty acid metabolism as acyltransferase cofactor and in energy production processes, such as interconversion in the mechanisms of regulation of ketogenesis and thermogenesis. Therefore, lack of l-carnitine leads to lipid accumulation in the cytosol and impaired energy production from long-chain fatty acids, especially during periods of fasting or stress. l-Carnitine pharmaceutical preparations, including injections, syrups, tablets, and capsules, are used in the therapy of primary and secondary carnitine deficiency, and in other diseases such as dislipoproteinemia and Alzheimer's [1–4].

A detailed literature survey revealed that there are few analytical methods reported for the estimation of l-Carnitine in pharmaceutical formulations. The US Pharmacopeia (USP) provides two HPLC methods for quantitation of l-carnitine in oral solution and tablet formulations. The method for tablets involves an aminopropylsilane-bonded silica gel column, acetonitrile-phosphate buffer (pH 4.7) mobile phase, and detection at 205 nm. This method requires a prolonged equilibration of the column (6 h), which is therefore time consuming in case the formulation contains an organic acid, due to the long retention time of the acid under the specified HPLC conditions [5]. On the other hand, for solution formulations, USP presents an HPLC method using ion-pairing modifiers. However, this method cannot separate crotonoylbetaine (impurity A) (Figure 1(b)), a major impurity and degradation product, from l-carnitine [6]. Other reported methods for quantification of l-carnitine in tablets are limited in either low sensitivity for dissolution testing or not being stability-indicating [5–8].

Environmental conditions, including light, heat, and humidity and the susceptibility of the substance towards hydrolysis or oxidation can play an important role in the production of impurities. A stability study ensures the maintenance of product quality, safety, and efficacy throughout its shelf life. Stress testing can help identify degradation products and provide important information about the intrinsic stability of drug substances [9, 10]. Regulatory agencies recommend the use of stability-indicating assay methods for the analysis of stability samples [11]. With the advent of the International Conference on Harmonization (ICH) guidelines [12, 13], requirements for the establishment of stability-indicating assay methods have become more clearly mandated [14].

Taking ICH guidelines into consideration, the present study describes a simple, validated, and stability-indicating analytical method for determination of l-carnitine in tablets. Also, the calculation of the measurement uncertainty which is based on the validation of the analytical procedures in a laboratory is presented. Moreover, the performances of the method were evaluated and its potential for the determination of l-carnitine in tablets was investigated.

## 2. Experimental Section

### 2.1. Materials, Reagents, and Chemicals

Qualified l-carnitine l-tartrate standard (99.37% equivalent to 67.79% l-carnitine) and crotonoylbetaine (impurity A) were kindly provided by Poursina pharmaceuticals (Tehran, Iran). Absolute ethanol, gradient grade methanol, and analytical grade reagents were purchased from Merck (Darmstadt, Germany). HPLC-grade water was obtained through a Milli-Q system (Millipore, Milford, MA, USA) and was used to prepare all solutions. The placebos (mixture of all the excipients according to RxList [15]) were prepared in our laboratory. l-Carnitine tablets (250 mg) manufactured by Shahrdarou Pharmaceuticals Ltd. (Iran) was purchased from local pharmacy.

### 2.2. Preparation of Standard and System Suitability Solutions

Stock standard solution of l-carnitine was prepared in water at a concentration of 67.79 mg/mL. Freshly prepared working standards at concentration levels of 84.74, 169.48, 338.95, 677.90, 1355.80, and 3389.50 *µ*g/mL were obtained from stock solution by the appropriate dilution in HPLC-grade water. For system suitability solution, accurately weighed quantities of l-carnitine and crotonoylbetaine were dissolved in water to obtain a solution having concentrations of 1500.00 and 7.00 *µ*g/mL, respectively.

### 2.3. Preparation of Test Solutions

Ten tablets' (l-carnitine label claim: 250 mg per tablet) content was weighed and the average weight of each tablet was calculated. Tablet powder equivalent to 250 mg of the active pharmaceutical ingredient was transferred into a 25 mL volumetric flask. To this, 15 mL of water was added and sonicated for 10 minutes. The solution was then diluted to 25 mL with diluent and centrifuged at 3000 rpm for 10 min. Two mL of the supernatant was transferred into a 10 mL volumetric flask. Thereafter, the volume was adjusted to the mark with the same medium to provide a theoretical concentration of 2.00 mg/mL of l-carnitine. The experiment was performed by triplicate. The l-carnitine dissolution profiles were studied in marketed tablets (250 mg), and the measurements were obtained using the paddle apparatus described in Method 2 of USP. The agitation speed used was 75 rpm, which are the recommended conditions for dissolution methods applied in USP l-carnitine monograph. The volume of water as dissolution media was 900 mL, maintained at 37 ± 0.5°C. Sampling was performed manually at the following time points, 10, 15, 20, and 30 min. These samples were assayed using calibration curves of working standard solutions.

### 2.4. HPLC Analysis

The HPLC method was carried out on a Younglin (Hogye, Republic of Korea), set to recycle the mobile phase and was equipped with YL9104 Vacuum degasser, YL9110 Quaternary pump, YL9131 Column compartment, and YL9120 UV/VIS detector. The peak areas were integrated automatically by computer using an Autochro-3000 software program. A 20 *µ*L volume of sample was introduced into a Rheodyne model 7725i injector.

The elution was carried out on a C18 column (250 mm × 4.6 mm, 5 *µ*m particle size) from Teknokroma (Barcelona, Spain). All analyses were performed at the column temperature of 50 ± 1°C under isocratic conditions with a mobile phase of 0.05 M phosphate buffer (pH = 3): ethanol (99 : 1), including 0.56 mg/mL of sodium 1-heptanesulfonate and a flow rate of 2.0 mL/min, using UV detection at 225 nm.

### 2.5. Forced Degradation Studies

The stability-indicating capability of the method was determined by subjecting l-carnitine solutions (standard and pharmaceutical preparations) at the concentration level of 6.78 mg/mL to accelerated degradation by acidic, basic, heat, oxidative, and photolytic conditions to evaluate the interferences in the quantitation of l-carnitine. Sample solutions prepared in 1 M hydrochloric acid and 1 M sodium hydroxide were used for the acidic and basic hydrolysis, respectively. Both solutions were heated at 70°C for 12 h and then neutralized with basic or acidic solutions, as necessary. For evaluating the heat condition, the sample solutions heated at 70°C for 12 h. For oxidative degradation, sample solutions were exposed to a solution of hydrogen peroxide (3%) and kept at ambient temperature for 4 h, protected from light. Photodegradation was induced by exposing the sample solution to UV-Lamp at a wavelength of 254 nm placed in a wooden cabinet for 4 hours. The experiments were performed in triplicate. The solutions were diluted with HPLC-grade water to final concentration of 1355.80 *µ*g/mL and were injected into chromatograph.

### 2.6. Method Validation

The developed method was validated as per the requirements of the ICH guidelines. Linearity was evaluated by determining six working standard solutions at a concentration range of 84.74–3389.50 *µ*g/mL. Five sets of such solutions were prepared. Each set was analyzed to plot a calibration curve. Slope, intercept and coefficient of determination (*r* ^2^) of the calibration curves were calculated to ascertain linearity of the method.

The limit of quantification (LOQ) was defined as the lowest concentrations with the RSDs lower than 5% and accuracies within ±5%, considering at least ten times the response compared to that of the blank.

In order to check the robustness, the effect of small but deliberate variations in the chromatographic conditions was evaluated. The conditions studied were flow rate (altered by ±0.2 mL/min), column temperature (altered by ±2°C), and pH of phosphoric acid solution (altered by ±0.1). These chromatographic variations were evaluated for resolution between l-carnitine and crotonoylbetaine, % assay of the drug, and theoretical plates and tailing factors of the peaks.

For method repeatability, assay of working standard solutions (84.74, 169.48, 1355.80, and 3389.50 *µ*g/mL) was repeatedly performed five times on the same day (intraday). For reproducibility, freshly prepared solutions at aforementioned concentration levels were analyzed at different days (interday) and results were statistically evaluated in terms of % RSD.

For recovery studies, preassayed portions of powdered tablets equivalent to 250 mg of l-carnitine were spiked with extra 0.25, 0.50, and 0.75 mL of a solution of 100 mg/mL of l-carnitine. These samples were handled as explained in sample preparation section and the final target levels of 2.20, 2.40, and 2.60 mg/mL were prepared. The concentrations were calculated using calibration curves.

Accuracy was calculated as the deviation of the mean from nominal concentration. To assess accuracy, freshly prepared placebo of the l-carnitine pharmaceutical tablets was spiked with various amounts of l-carnitine to obtain the concentration levels of 84.74, 169.48, 1355.80, and 3389.50 *µ*g/mL. Each solution was injected by triplicate.

### 2.7. Estimation of the Uncertainty of the Measurements

An expanded uncertainty budget was constructed for l-carnitine in pharmaceutical preparations by the RP-HPLC-UV method according to previously reported procedures [16].

Four individual sources including uncertainties associated to the measurement standard, calibration curve, precision, and accuracy were taken into account to assess the expanded uncertainty.

## 3. Results and Discussion

### 3.1. Optimization of the Chromatographic Conditions

The HPLC procedure was optimized with a view to develop a stability-indicating HPLC method with a short run time while keeping the system suitability necessities according to USP, which needs the resolution between l-carnitine and impurity A and the relative standard deviation for replicate injection to be greater than 1.0 and less than 2.0%, respectively. Moreover, the method should be sensitive enough to be able to estimate dissolution profile of the tablets.

Initial effort for the method development considering the high polarity of the analytes was made using USP mobile phase for determination of oral solutions in a common ODS column with 250 mm length. Under this condition no separation was achieved between l-carnitine and impurity A. Different pH(s) were screened by adjusting the pH of the buffer. Optimum resolution was observed at pH 3.0. However, the peaks were tailed and the resolution between the analytes did not meet the USP requirement. In our previous experiment with diltiazem analysis, which is a tertiary amine, we realized that using ethanol as organic modifier in mobile phase provided sharper peaks and better resolution between diltiazem and its impurity [16]. Therefore, ethanol was used instead of methanol in the composition of the mobile phase, which led to sharper peaks compared to a mobile phase consisting of methanol but the analytes were not well resolved. Thereafter, optimization of ethanol content in mobile phase, column temperature, and flow rate was performed and the best peak shapes and resolution were obtained when the aforementioned parameters were set at 1.0%, 50.0°C, and 2.0, respectively. Considering low UV absorptivity of carnitine, wavelength of 225 nm was chosen to have suitable sensitivity.

**Figure 2:**
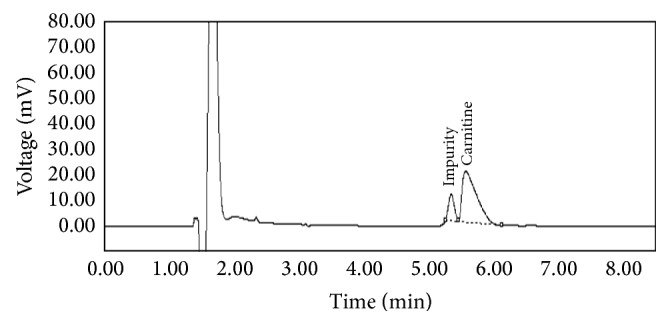
Typical chromatogram of l-carnitine and its main impurity (crotonoylbetaine).

Under the chromatographic conditions of this method, the resolution between crotonoylbetaine and l-carnitine was 1.1 ± 0.9% (Figure 2), the theoretical plates for l-carnitine peak was 2087.0 ± 0.82%, the tailing factor for l-carnitine peak was 1.3 ± 1.54%, and total run time was less than 8 min. Before being fully implemented in the quantitative determination of drug substance and pharmaceutical preparation, this method was thoroughly validated according to ICH guidelines.

### 3.2. Forced Degradation Studies

**Figure 3:**
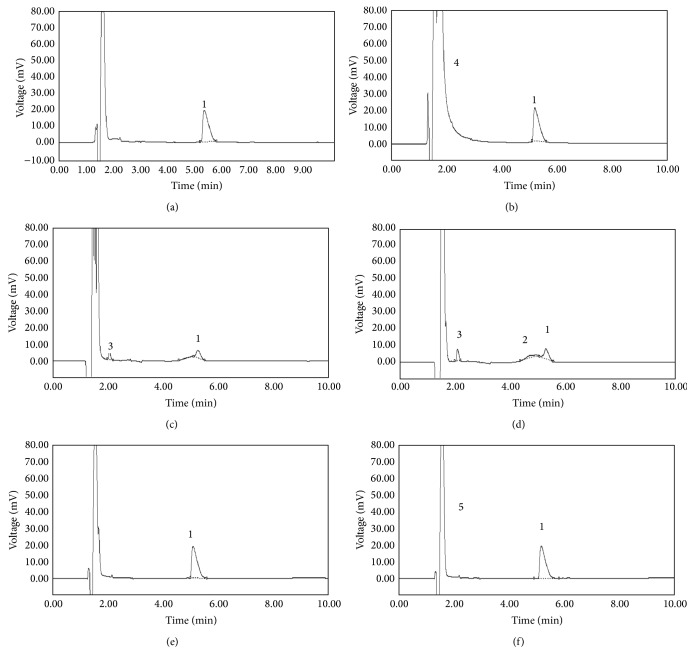
Typical chromatograms of l-carnitine after degradation under (a) photolytic condition; (b) oxidative condition: peak 4 = hydrogen peroxide; (c) basic hydrolysis; peak 3 = unknown impurity (d); acidic hydrolysis: peak 3 = unknown impurity, peak 2 = Impurity A; (e) heat condition; and (f) l-carnitine working standard solution (1355.80 *µ*g/mL) Peak 1 = l-carnitine, and peak 5 = tartaric acid.

The main criterion for developing a stability-indicating HPLC method for determination of l-carnitine was to be accurate and free of interference from other degradation products, process impurities, excipients, or other potential impurities and convenient enough for routine use in quality control laboratories. l-Carnitine showed drastic degradation in acidic and basic conditions, in the fact that only 24.0% ± 0.81 and 17.35% ± 1.72 of the drug remained, respectively, and at the same time an additional peak was detected at 2 min (Figures 3(c) and 3(d)). The forced degradation studies in photolytic, heat, and oxidative conditions, resulted in nonsignificant decrease of the area without any detectable eluting degradation product. Under these conditions, 99.50% ± 0.63, 93.09% ± 1.93, and 100.17% ± 1.28 of l-carnitine were recovered, respectively (Figures 3(a), 3(e), and 3(b)). The degradation products of the parent compound were found to be similar for both the pharmaceutical and standard solutions. All the degradation studies are summarized in Table 1.

### 3.3. Method Validation

#### 3.3.1. Specificity

Specificity is the ability of the method to unequivocally assess the analyte response in the presence of its potential impurities that was illustrated by the acceptable separation of l-carnitine from degradation products as shown in Figure 3. Furthermore, the decreases observed in l-carnitine contents in stability studies, when degradation products appeared, proved the specificity of the method (Table 1). Consequently, the forced degradation studies documented the stability indicating power and specificity of the proposed method.

#### 3.3.2. Linearity, Precision, and LOQ

Linearity was determined by constructing five independent calibration curves, each with six calibration points of l-carnitine, including the LOQ, in the range of 84.74–3389.50 *µ*g/mL. The peak areas of l-carnitine against the respective concentrations were used for plotting the graph, and the linearity was evaluated by the least square regression analysis. The linearity curve was defined by the following equation: *y* = 0.19*x* + 2.31 (*r* ^2^ = 0.9997) which indicated the linearity of the calibration curve for the method. Moreover, the relative standard error of slope can be used as a parameter with respect to the precision of the regression, as a general acceptance criterion for the linearity performance of the analytical procedure [17]. This parameter should be comparable to the calculated RSD in the evaluation of the precision. In this study, the result obtained for the RSD of the slopes was 1.92% which is comparable to mean value 1.56%, of the RSD of the precision.

Summary of the method validation results is shown in Table 2. The method was proved to be precise, as the intra- and interday precision calculated for the concentration levels of 169.48, 1355.80, and 3389.50 *µ*g/mL ranged from 0.41% to 1.84% and 0.99% to 1.60%, respectively. These values fulfill the validation criteria of an analytical method designed for quality control of pharmaceutical preparations for which RSD values < 2% are acceptable [17].

The LOQ is the lowest concentration that can be quantified with acceptable precision and accuracy. The LOQ of l-carnitine was determined to be 84.74 *µ*g/mL, considering the mean accuracy value of 97.27% and RSD value of 3.34% (Table 2). These values indicate that the proposed method is more sensitive than what have reported previously for analysis of l-carnitine in tablet formulations using UV detection (LOQ = 400 *µ*g/mL) [6]. The theoretically “expected” concentration of l-carnitine after the dissolution experiments is ca.277.78 *µ*g/mL (250 mg l-carnitine per tablet in 900 mL dissolution medium assuming quantitative dissolution). The LOQ obtained in this study was 30% of the target concentration and brackets effectively the abovementioned concentration.

#### 3.3.3. Recovery and Accuracy

The accuracy was evaluated applying the proposed method to the analysis of the in-house mixture of the tablet excipients with known amounts of the drug, to obtain solutions at concentration levels of 84.74, 169.48, 1355.80, and 3389.50 *µ*g/mL. The accuracy was assessed from three replicate determinations and calculated as the percentage of the drug recovered from the formulation matrix. The mean and RSD values calculated for the analysis of three l-carnitine concentration levels of 169.48, 1355.80, and 3389.50 *µ*g/mL are shown in Table 2; the mean values were found to be 100.72%, 98.52%, and 99.51% with RSDs 0.75%, 0.83%, and 1.05%, respectively, demonstrating that the method is accurate within the desired range. Also, the results obtained from the analysis of preassayed tablets spiked with different amounts of l-carnitine stock solution revealed acceptable recoveries with the mean value of 101.09 and % RSDs < 1.80, respectively. These values document a high recovery in this method.

#### 3.3.4. Robustness

Chromatographic parameters including percentage of assay, resolution between l-carnitine and its impurity, theoretical plates, and tailing factor of l-carnitine peaks were not significantly affected by the slight changes in the chromatographic conditions like alteration in flow rates, pH of the aqueous solution of mobile phase, and column temperature. Analysis was carried out in triplicate and only one parameter was changed in the experiments at a time. The results of the experimental variables evaluated were within the acceptable deviation (RSD < 2%), the resolution of the aforementioned peaks was more than 1.0, and the theoretical plates and tailing factor parameters were calculated to be more than 2000 and less than 1.4, respectively, indicating that the proposed method is robust under the conditions tested.

#### 3.3.5. The Uncertainty of the Method

The expanded uncertainty of the method for quantification of l-carnitine in pharmaceutical preparations was calculated to be 0.06 mg/mL. Partial (*U*_standard_, *U*_calibration_, *U*_precision_, and *U*_accuracy_) and expanded uncertainties associated with the analytical results (expressed as % relative standard deviation) were estimated to be 0.36%, 0.33%, 1.07%, 1.04%, and 3.14%, respectively. The concentration of sample assayed was 1.98 mg/mL. Among the four sources of uncertainty, which were taken into consideration, the uncertainty associated with precision appears to be the most important source in the overall uncertainty. Therefore, analysts should pay great attention when performing such experiments.

#### 3.3.6. Application of the Method

**Figure 4:**
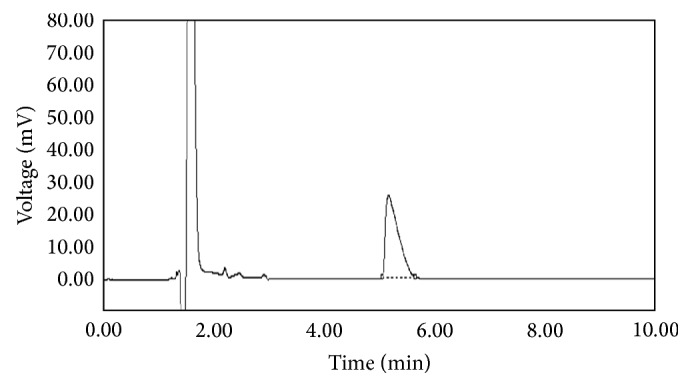
A chromatogram obtained from analyzing of the commercially available tablets.

**Figure 5:**
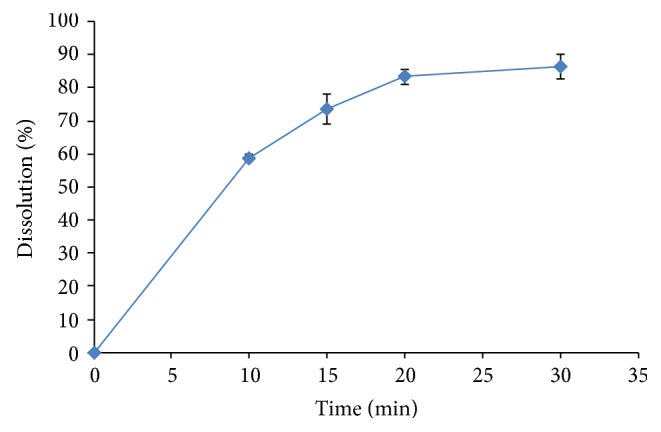
Dissolution profile of l-carnitine in commercial tablets (*n* = 6).

The optimized and validated method was applied for the determination of l-carnitine in marketed tablets. The amount of l-carnitine in tablets and also their dissolution profiles were quantitative estimated using calibration curve method. Typical chromatogram and dissolution profile obtained following the assay and dissolution testing of the pharmaceutical dosage form are shown in Figures 4 and 5, respectively. The value of 99.00% of label claim indicates that the method is selective for the analysis of l-carnitine without interference from the excipients used to formulate and produce these tablets. Moreover, the method is rapid and sensitive enough to evaluate the dissolution of l-carnitine tablets.

## 4. Conclusion

The stability-indicating and rapid RP-HPLC method developed for the quantitative analysis of l-carnitine in pharmaceutical dosage forms is precise, linear, accurate, specific, and robust. To the best of our knowledge, this is the first method which reports the metrological parameters in quantification of l-carnitine in pharmaceutical tablets. In addition, recycling significantly reduced the mobile phase consumption and made the method economic. Moreover, the method is more sensitive than the previously reported procedure [6].

Finally, the improved method was successfully performed to the analysis of l-carnitine in pharmaceutical tablets and it can thus be used for routine analysis, quality control, and studies of the stability of tablets containing l-carnitine.

## Figures and Tables

**Table 1:** Summary of stress degradation studies of L-carnitine.

Stress condition/media/duration	Recovered l-carnitine (%) mean (RSD, %)	Number of observed impurities
Photolytic/H_2_O/254 nm/4 h	99.50 (0.63)	0
Acidic/1.0 N HCl/70°C/12 h	24.00 (0.81)	2
Neutral/H_2_O/70°C/12 h	93.09 (1.93)	0
Oxidative/3.0% H_2_O_2_/4 h	100.17 (1.28)	0
Basic/1.0 N NaOH/70°C/12 h	17.35 (1.72)	1

**Table 2:** Precision, accuracy, and recovery data for the proposed method.

L-Carnitine concentration (*µ*g/mL)	Precision (RSD, %)		Accuracy (*n* = 3) mean (RSD, %)	Recovery (*n* = 3)			
	Intraday (*n* = 5)	Interday (*n* = 5)		Target concentration (mg/mL)	Calculated concentration mg/mL (mean)	Percentage	±SD
84.74	3.34	2.18	97.27 (2.38)	2.20	2.22	100.91	0.04
169.48	0.41	1.36	100.72 (0.75)	2.40	2.42	100.83	0.01
1355.80	0.83	0.99	98.52 (0.83)	2.60	2.64	101.54	0.02
3389.50	1.84	1.60	99.51 (1.05)				
